# Targeting the Gut–Heart Axis in Diabetic Heart Failure: Microbiota and SGLT2is as Converging Therapeutic Frontiers

**DOI:** 10.3390/ijms27094101

**Published:** 2026-05-03

**Authors:** Yen Chu, Kuo-Hsiung Huang, Chi-Nan Tseng

**Affiliations:** 1 Division of Thoracic and Cardiovascular Surgery, Chang Gung Memorial Hospital Linkou Branch, Taoyuan 33305, Taiwan; 2 Laboratory of Cardiovascular Physiology, Chang Gung Memorial Hospital Linkou Branch, Taoyuan 33305, Taiwan; 3 Department of Research and Development, Chang Gung Memorial Hospital Linkou Branch, No. 5, Fuxing St., Guishan Dist., Taoyuan 33305, Taiwan; 4 Department of Nursing, College of Nursing, Chang Gung University of Science and Technology, No. 261, Wenhua 1st Rd., Guishan Dist., Taoyuan 33303, Taiwan; khs586@cgmh.org.tw; 5 Graduate Institute of Traditional Chinese Medicine, The Medical College, Chang Gung University, No. 259, Wenhua 1st Rd., Guishan Dist., Taoyuan 33302, Taiwan; 6 Department of Laboratory Medicine, Section of Clinical Serology and Immunology, Chang Gung Memorial Hospital, Linkou Branch, Taoyuan 33305, Taiwan

**Keywords:** gut–heart axis, diabetic heart failure, gut microbiota, sodium glucose cotransporter 2 inhibitors (SGLT2is), dysbiosis, trimethylamine N-oxide (TMAO), short-chain fatty acids (SCFAs), farnesoid X receptor (FXR), Takeda G protein-coupled receptor 5 (TGR5), endothelial dysfunction, bile acid signaling, ketone metabolism, microbiome profiling, cardiometabolic remodeling, personalized therapy

## Abstract

Emerging evidence highlights the gut microbiota as a critical modulator in the pathogenesis of heart failure (HF), particularly among patients with type 2 diabetes mellitus (T2DM). Dysbiosis contributes to systemic inflammation, endothelial dysfunction, and adverse cardiac remodeling via microbial metabolites such as trimethylamine N-oxide (TMAO) and short-chain fatty acids (SCFAs). However, the therapeutic intersection between the gut microbiota and pharmacological interventions remains insufficiently integrated. Sodium-glucose cotransporter-2 inhibitors (SGLT2is), a cornerstone of T2DM management, confer cardioprotective effects that may involve microbiota-mediated pathways. This review provides a novel synthesis of how SGLT2is influence gut ecology, specifically through altered glucose excretion and osmotic shifts, to potentially restore SCFA-producing taxa. By delineating the structural transitions from gut physiology to SGLT2i-modulated cardiac outcomes, we emphasize the gut–heart axis as a pivotal therapeutic target. This focused framework offers new insights into the triadic interplay between microbiome stability and cardiometabolic health, moving beyond traditional glucose-centric paradigms.

## 1. Introduction

### 1.1. Brief Overview of HF in T2DM Patients

HF is a major cardiovascular complication in patients with T2DM, with prevalence and incidence rates significantly higher than in the general population [1]. Epidemiological studies confirm that T2DM confers a markedly increased risk of HF, highlighting the clinical burden of this comorbidity [2].

The pathophysiology of HF in T2DM is multifactorial. Chronic hyperglycemia, insulin resistance, and metabolic derangements contribute to endothelial dysfunction, microvascular impairment, and systemic inflammation [3]. These processes accelerate myocardial fibrosis, impair calcium handling, and alter substrate utilization, ultimately predisposing patients to both HF with reduced ejection fraction (HFrEF) and HF with preserved ejection fraction (HFpEF) [4]. Recent advances in integrative metabolomics and genomics have accentuated novel pathways linking metabolic dysregulation to cardiac remodeling in T2DM, offering insights into potential therapeutic targets [5]. In addition, reduced exercise tolerance has been consistently observed in diabetic patients, reflecting impaired cardiopulmonary reserve and contributing to poor prognosis [6].

HF in T2DM represents a complex interplay of metabolic, vascular, and functional abnormalities. Despite therapeutic progress, this dual burden remains a major challenge, reinforcing the need for comprehensive strategies that address both glycemic control and cardiovascular protection [1,2,3,4,5,6].

### 1.2. Role and Therapeutic Impact of SGLT2is

SGLT2is have emerged as keystone agents in the management of patients with T2DM and HF, demonstrating consistent reductions in cardiovascular mortality and hospitalization across heterogeneous cardiometabolic phenotypes [7]. In a large cohort study involving 1130 individuals with HF and reduced ejection fraction, SGLT2i therapy significantly improved metabolic indices and attenuated adverse cardiac outcomes independent of glycemic status [8]. Meta-analyses of multinational randomized trials further substantiate the cardiorenal protective effects of SGLT2is, which extend beyond glycemic modulation to encompass natriuresis, enhanced myocardial energetics, and suppression of systemic inflammation [9]. Among patients presenting with acute myocardial infarction and concomitant T2DM, SGLT2i use was associated with reductions in HF hospitalization and a lower incidence of HF onset, emphasizing their utility in secondary prevention [10]. These salutary outcomes have catalyzed the formal integration of SGLT2is into contemporary HF management guidelines, extending their therapeutic indication to encompass both diabetic and non-diabetic cohorts [7,10].

### 1.3. Emerging Relevance of Gut Microbiota in Cardiovascular Health

The gut microbiota has emerged as a crucial modulator of cardiometabolic homeostasis, with accumulating evidence linking microbial composition and metabolite profiles to the pathogenesis and progression of diabetic HF [11]. Dysbiosis-driven alterations in SCFAs, TMAO, and bile acid signaling have been implicated in endothelial dysfunction, myocardial fibrosis, and systemic inflammation, hallmarks of diabetic cardiomyopathy [12,13]. Recent cohort and multiomics studies highlight the mechanistic relevance of the gut–heart axis, including the Framingham Heart Study profiling, which revealed that cholesterol-metabolizing bacterial taxa associate with cardiovascular risk markers [12]. Mechanistic investigations reveal that gut butyrate producers confer cardiac protection by modulating systemic inflammatory responses [14], while clinical data from TAVI patients demonstrate that elevated levels of indoxyl sulfate and TMAO significantly correlate with impaired left ventricular function [15]. Collectively, the gut microbiota modulates host cardiometabolic homeostasis through specific metabolite signaling axes. These pathways are central to the pathogenesis of T2DM-associated HF. This evidence supports a novel triadic model. In this framework, microbial composition determines metabolite output, while these metabolites subsequently alter vascular and myocardial biology. Furthermore, the underlying T2DM amplifies overall cardiac vulnerability. Crucially, SGLT2is serve as a central pharmacologic interceptor within this triad; multiomics profiling elucidates that these agents not only mitigate myocardial metabolic stress and inflammatory signaling [16,17], but also alter gut microbial composition and bile acid pools to influence farnesoid X receptor (FXR) and TGR5 activation [18]. Together, these data identify specific mechanistic nodes, such as TMA lyases; SCFA pathways; BA receptors, including FXR and Takeda G protein-coupled receptor 5 (TGR5); and NLRP3 or TLR4 inflammatory axes, as actionable targets for translational intervention. The scope of this review is to elucidate the precise mechanistic pathways linking microbial metabolites to myocardial dysfunction while evaluating how SGLT2i and microbiota-directed therapies converge on these critical therapeutic nodes.

### 1.4. Search Strategy and Selection Criteria

This narrative review was conducted by searching PubMed and ScienceDirect for publications through March 2026. We prioritized peer-reviewed randomized controlled trials, prospective cohort studies, systematic reviews, and high-quality preclinical investigations that addressed microbiota composition, microbial metabolites, and cardiometabolic outcomes and to provide a conceptual synthesis of emerging paradigms, emphasizing mechanistic and translational insights.

Search queries combined terms including “microbiota”, “type 2 diabetes mellitus”, “SGLT2i”, “gut–heart axis”, “gut kidney axis”, “cardiometabolic health”, and “heart failure”. We excluded case reports, conference abstracts without full text, and non-peer-reviewed preprints. Only English-language articles were considered. The final selection emphasized mechanistic insights, clinical outcomes, and translational implications, thereby providing a comprehensive synthesis of current evidence on the microbiota–SGLT2i–HF axis. For transparency, we provide a Supplementary Table (Table S1) that summarizes study design, population or model with reported effect sizes, principal findings, and a brief risk of bias assessment.

## 2. Gut Microbiota and Cardiovascular Physiology

### 2.1. Composition and Function of Healthy Gut Microbiota

In the eubiotic state, the healthy gut microbiota is characterized by a balanced and diverse ecosystem predominantly composed of the phyla Firmicutes, Bacteroidetes, Actinobacteria, and Proteobacteria. These core microbial communities function symbiotically to maintain intestinal barrier integrity, regulate host immune homeostasis, and facilitate complex carbohydrate fermentation [18]. The resulting production of beneficial metabolites ensures an effective defense against pathogen colonization and preserves optimal metabolic function prior to the onset of any pathological alterations. Given that thousands of microorganisms inhabit the human gut, which covers a functional surface area of approximately 30 to 40 m^2^ this intricate population is now recognized as indispensable for maintaining systemic homeostasis and overall health [19].

Perturbations in microbial populations, termed dysbiosis, compromise host metabolic integrity and have been increasingly implicated in the pathogenesis of cardiovascular disease (CVD) [20]. A growing body of evidence now demonstrates that gut-derived metabolites exert profound effects on cardiovascular physiology and disease progression. Key bioactive molecules, including SCFAs, trimethylamine (TMA) and its oxidized derivative TMAO, BAs, and lipopolysaccharides (LPS), have been mechanistically linked to hypertension, atherosclerosis, and myocardial dysfunction [21,22]. These metabolites influence cardiovascular outcomes through diverse pathways ranging from modulation of microbial community structure to activation of host signaling cascades that govern endothelial function, vascular inflammation, and myocardial energetics [22].

Recent profiling from the Framingham Heart Study demonstrated that cholesterol-metabolizing taxa were independently associated with cardiovascular risk markers, emphasizing the systemic relevance of microbial composition [12]. Complementary cohort analyses confirm that enrichment of SCFA-producing genera correlates with improved vascular function and reduced cardiometabolic risk [23,24]. Gut microbiota imbalance, or dysbiosis, correlates with reduced microbial diversity and increased pathobiont abundance, which are associated with impaired vascular reactivity and higher HF hospitalization risk in large prospective cohorts [25,26]. Multi-cohort analyses show that dysbiosis signatures predict adverse remodeling and decreased peak oxygen consumption (VO_2_) in HF patients, linking microbial composition directly to functional limitation and outcomes [27,28]. Longitudinal cohort data further demonstrate that shifts toward SCFA-depleted and TMAO-enriched profiles track with worsening cardiac function and increased inflammatory biomarkers, reinforcing causally relevant pathways in cardiovascular impairment [29,30].

### 2.2. Mechanisms Linking Dysbiosis to Endothelial Dysfunction, Inflammation, and HF Progression

Dysbiosis disrupts intestinal barrier integrity and promotes translocation of microbial metabolites, driving systemic inflammation and endothelial dysfunction [31]. A prospective cohort of newly diagnosed HF patients revealed that altered SCFA profiles were linked to impaired vascular responses and heightened inflammatory burden [32]. Larger multicenter analyses further highlight that gut microbiota dysregulation contributes to HFpEF progression via pro-inflammatory cytokines such as TNF-α and IL-6 and C-reactive protein (CRP) elevation, reinforcing the mechanistic role of the gut–heart axis [32]. Cohort studies associate elevated gut-derived toxins, such as TMAO and indoxyl sulfate, with impaired endothelial-dependent vasodilation, greater arterial stiffness, and higher incident HF events [33,34]. Population-based data link dysbiosis-driven bile acid signaling perturbations to endothelial activation and microvascular inflammation, aligning with progressive diastolic dysfunction trajectories in HFpEF [16]. Integrated multi-omics cohorts reveal that microbiota-derived inflammatory signatures (e.g., TNF-α, IL-6, and CRP) mediate the relationship between dysbiosis and adverse cardiac remodeling, accelerating HF progression via endothelial injury and mitochondrial energetic stress [35,36].

### 2.3. Microbial Metabolites and Host Mechanisms in Diabetic HF

The gut microbiota produces a diverse array of bioactive molecules that serve as critical messengers in cardiovascular homeostasis [19]. Among these, SCFAs, TMAO, and bile acids represent the primary metabolic nodes linking gut dysbiosis to myocardial dysfunction. SCFAs such as butyrate, propionate, and acetate exert vital protective signals by acting as ligands for host receptors, including G protein-coupled receptor 41 and 43 (GPR41/43) [37,38,39,40]. These interactions promote vasoprotective and anti-inflammatory effects, which are essential for maintaining vascular compliance and suppressing chronic low-grade inflammation [41,42]. In the context of diabetic HF, the severe depletion of these metabolites removes these protective signals, thereby may contribute to increased vascular resistance and the progression of diabetic cardiomyopathy [42,43,44].

Conversely, the compromised intestinal barrier allows for the systemic translocation of microbial components, notably LPS, a cell wall component of Gram (−) bacteria [45]. The presence of these metabolites is linked to a heightened inflammatory milieu characterized by the activation of toll-like receptor 4 (TLR4) and tumor necrosis factor receptor signaling pathways [42,43,44]. Furthermore, specific microbial products such as TMAO may serve as robust independent predictors of incident heart failure and major adverse cardiovascular events in diabetic cohorts [46]. While preclinical models provide compelling evidence that elevated concentrations directly impair intracellular calcium handling and promote myocardial fibrosis, human data remain predominantly associative [47]. The convergence of these harmful metabolic signals with the loss of beneficial SCFAs creates a pathogenic environment that accelerates myocardial structural remodeling. The restoration of this metabolic balance through SGLT2i therapy, potentially facilitated by off-target SGLT1 inhibition in the proximal intestine [48], represents a promising frontier for mitigating the cumulative burden of microbial toxicity on the failing heart [49].

Understanding these fundamental gut-host interactions provides the necessary context to explore how their disruption contributes to specific disease states. In the following section, we detail how dysbiosis and metabolite imbalance drive the progression of diabetic HF.

## 3. Gut Microbiota in Diabetic HF

### 3.1. Dysbiosis and Systemic Inflammation

T2DM is consistently characterized by a state of dysbiosis defined by a significant reduction in microbial diversity and a detrimental shift in the functional capacity of the community [43]. This diabetic dysbiosis typically features a significant depletion of major SCFA-producing taxa, particularly the butyrate and propionate producers such as *Faecalibacterium prausnitzii* and *Roseburia* spp. [50]. The resulting lack of SCFA fuel is detrimental to the colonocytes, leading to impaired integrity of the tight junctions that form the epithelial barrier, creating a leaky gut [51]. This compromised barrier allows for the systemic translocation of LPS [52]. The resulting state, known as metabolic endotoxemia, shows elevated circulating LPS levels strongly associated with insulin resistance and the low-grade systemic inflammation characteristic of diabetic cardiomyopathy [53].

LPS functions as a potent pathogen-associated molecular pattern (PAMP) that engages TLR4 signaling in immune and endothelial cells. This activation drives a chronic pro-inflammatory state characterized by elevated TNF-α and IL-6, which are central mediators of adverse cardiac remodeling in T2DM. Recent cohort evidence has reinforced this mechanism. Pro-inflammatory macrophages were shown to release damage-associated molecular patterns (DAMPs) that activate TLR4 and TNFR signaling in cardiomyocytes, leading to sustained inflammation, caspase activation, and structural remodeling of the heart [37].

### 3.2. Impact on Endothelial and Myocardial Function

The cumulative burden of diabetic dysbiosis and the resulting metabolite imbalance directly impairs the structural and functional integrity of both endothelial and myocardial tissues. Endothelial dysfunction is severely exacerbated by metabolic endotoxemia and elevated levels of TMAO. Experimental evidence demonstrates that TMAO induces vascular inflammation by activating the NLRP3 inflammasome. This process occurs through the sirtuin 3 (SIRT3)–superoxide dismutase 2 (SOD2)–mitochondrial reactive oxygen species (mtROS) signaling axis and ultimately leads to increased vascular stiffness [54]. Within the myocardium, chronic low-grade inflammation is fueled by LPS. When coupled with the direct pro-fibrotic signaling of TMAO, these factors contribute to the pathological remodeling seen in diabetic cardiomyopathy. Original research has suggested that TMAO exacerbates cardiac fibrosis specifically by activating the NLRP3 inflammasome, facilitating the phenotypic transition of cardiac fibroblasts into active myofibroblasts [55]. Furthermore, the metabolic inflexibility of the diabetic heart is compounded by the deficiency of SCFA-mediated metabolic cues, resulting in an energy-starved myocardium [56].

While preclinical studies demonstrate that TMAO activates the NLRP3 inflammasome and promotes fibrosis [55], human data remain largely associative, such as correlations between plasma TMAO and adverse HF outcomes [57]. Proven clinical effects are limited to randomized trials of SGLT2 inhibitors showing reduced HF hospitalization [58]. This reframes the gut–heart axis as an emerging hypothesis rather than a validated therapeutic framework.

### 3.3. SGLT2 Inhibitor-Mediated Modulation of the Gut Microenvironment

Beyond their promising systemic glucose-lowering effects, SGLT2 inhibitors exert a distinct influence on the intestinal microenvironment by altering the availability of carbohydrate substrates in the distal gastrointestinal tract. This altered carbohydrate flux specifically enriches the population of *Bifidobacterium* and *Lactobacillus* species, while simultaneously suppressing the growth of proteolytic and pro-inflammatory taxa [59]. Consequently, SGLT2 inhibitors may act as indirect “prebiotics,” leveraging the gut–heart axis to restore metabolic homeostasis and mitigate the systemic inflammatory burden associated with diabetic HF. The induction of glucosuria by SGLT2is significantly alters intestinal osmolarity in addition to its systemic effects on glucose regulation. This shift in the luminal environment acts as a selective pressure that is less conducive to the proliferation of pathobionts [49]. Interestingly, specific agents such as canagliflozin have been shown to exhibit off-target inhibition of SGLT1 in the proximal intestine [48]. This dual mechanism further delays intestinal glucose absorption and significantly blunts postprandial glucose excursions, thereby potentially modulating the microbial landscape through altered nutrient availability [48].

Given the critical role of the gut–heart axis in HF, targeting this pathway has become a therapeutic priority. The following section examines how SGLT2is, a cornerstone of modern diabetic management, interact with the gut environment to exert cardio-protective effects.

## 4. SGLT2i-Microbiota Interactions: A Non-Glycemic Pathway to Cardioprotection

### 4.1. SGLT2is and the Gut–Microbiota–Metabolite Axis

SGLT2i treatment indirectly modulates the complex biotransformation of intestinal metabolites, particularly bile acids (BAs), via the gut microbiota. Recent multi-omics research utilizing 16S rRNA gene sequencing paired with LC-MS/MS profiling has demonstrated that SGLT2 inhibition alters the abundance of specific microbial taxa—such as the enrichment of *Lachnospiraceae* and *Muribaculum*—and significantly restructures the intestinal BA profile [37]. Crucially, these microbiota-driven shifts in the intestinal metabolome are tightly coupled with an enhanced secretion of glucagon-like peptide-1 (GLP-1), an incretin hormone renowned for its metabolic and cardioprotective effects [37].

### 4.2. Altered Glucose Flux and Osmolarity as Drivers of Dysbiosis Reversal

The increased delivery of glucose into the lower gastrointestinal tract fundamentally alters the nutritional landscape for the luminal microbiota [59]. Preclinical studies indicate that SGLT2i treatment leads to a favorable restructuring of the microbial community, characterized by the enrichment of SCFA-producing bacteria [38]. This enrichment is significant as it directly counters the SCFA depletion observed in diabetic dysbiosis [47]. Concurrently, SGLT2is reduce pathobionts linked to inflammation and TMA production [60]. Beyond glucose delivery, SGLT2i-induced glucosuria alters intestinal osmolarity, acting as a selective pressure that creates an environment less conducive to the growth of pathobionts [49].

### 4.3. Modulation of Bile Acid Signaling and Metabolite Pathways

SGLT2i therapy influences the composition of the BA pool circulating in the enterohepatic system [38]. By altering the microbial population, SGLT2is indirectly affect the enzymatic biotransformation of primary BAs into secondary BAs, which act as ligands for host nuclear receptors [37]. For example, changes in BA composition modulate the activation of the FXR in the ileum [39]. Likewise, the altered BA profile can enhance signaling through TGR5, which promotes anti-inflammatory effects and energy expenditure [40]. Furthermore, preclinical and limited clinical data suggest that SGLT2i treatment leads to a measurable reduction in circulating plasma TMAO levels, correlated with improved cardiac function [61]. Collectively, SGLT2is act as indirect microbiome modulators in both murine and human cohorts [62].

### 4.4. Effects on Microbial Composition and Diversity

The systemic effects of SGLT2is lead to measurable alterations in overall diversity [41]. A consistent finding is the modulation of the *Firmicutes* to *Bacteroidetes* ratio, a commonly cited metric of gut health [38]. At the genus level, SGLT2is promote the enrichment of beneficial taxa, such as SCFA producers and *Akkermansia muciniphila* [42,63]. Conversely, SGLT2i treatment suppresses Gram-negative bacteria within the *Proteobacteria* phylum, thereby mitigating the metabolic endotoxemia that drives systemic inflammation [41].

### 4.5. SGLT2i Versus Other HF Therapies: Microbiota Effects and Evidence Gaps

Compared with other guideline-directed HF therapies, evidence that SGLT2is reproducibly and directly remodel the gut microbiota is limited. The available data come mainly from preclinical models and small short-term human cohorts rather than from prospective head-to-head clinical comparisons with angiotensin-converting enzyme inhibitors (ACE inhibitors)/angiotensin II receptor blockers (ARBs), angiotensin receptor-neprilysin inhibitors (ARNIs), beta-adrenergic blockers, mineralocorticoid receptor antagonists (MRAs), or glucagon-like peptide-1 receptor agonists (GLP-1 receptor agonists) [40,64,65]. Most human studies are short-term and observational, so durable effects on microbial community structure and on longitudinal metabolite trajectories such as TMAO and SCFAs remain unproven in adequately powered clinical trials [37,40,61]. Whether SGLT2i–microbiota interactions differ by HF phenotype, for example, HfpEF versus HfrEF, has not been systematically evaluated despite mechanistic reasons to expect phenotype-specific effects on inflammation, diastolic mechanics, and myocardial substrate use [29,58]. Preclinical and small clinical reports describe SGLT2i-associated shifts in luminal nutrient availability and microbial composition that may favor SCFA-producing taxa, but these findings require replication in larger geographically diverse cohorts using standardized microbiome methods [37,65]. Substantial biogeographical and methodological heterogeneity, including dietary patterns, sequencing platforms, and bioinformatic pipelines, further limits generalizability and supports the need for long-term phenotype-stratified randomized trials with integrated microbiome and metabolite endpoints [62,66].

## 5. Microbiota–SGLT2i–HF Axis: Mechanistic Insights

### 5.1. Microbial Shifts and Enhanced Myocardial Energetics via Ketone Metabolism

SGLT2i treatment promotes a beneficial shift in myocardial substrate utilization, favoring ketone body oxidation [61]. By promoting mild ketogenesis, SGLT2is provide the failing myocardium with β-hydroxybutyrate (βOHB), a more efficient energy substrate [61]. The SGLT2i-induced shift toward an SCFA-rich microbial environment may act with systemic effects to boost circulating βOHB levels, fueling the energy-starved myocardium [41,67].

### 5.2. Integrated Pathways Linking Microbial Signaling and Vascular Tone

The beneficial impact of SGLT2is on the gut microbiome extends directly to the vascular endothelium, addressing the hallmark endothelial dysfunction of diabetic HF. This convergence primarily occurs through the regulation of key microbial metabolites. High plasma TMAO accelerates endothelial dysfunction and systemic inflammation, and studies have repeatedly shown that SGLT2i therapy not only reduces TMAO levels by reshaping the gut environment but also mitigates the proinflammatory and pro-atherogenic effects of existing TMAO, contributing to improved vascular tone and reduced arterial stiffness [40,68].

The SGLT2i-induced activation of FXR and TGR5 provides a marked anti-inflammatory pathway [38,39]; these molecular improvements likely contribute to the significant reduction in HF hospitalizations and renal progression observed in real-world patient cohorts [63]. While SGLT2i therapy significantly optimizes cardiac energy metabolism by shifting fuel utilization toward ketones [67], it simultaneously strengthens the intestinal barrier via FXR activation [38]. This dual action reduces the influx of inflammatory LPS, thereby attenuating the chronic low-grade inflammation that drives cardiac remodeling. Similarly, activation of TGR5, particularly on immune cells, directly dampens inflammatory responses [39,55]. Finally, the SGLT2i-driven increase in SCFAs promotes vasodilation and anti-inflammation by activating host receptors, specifically GPR43 and GPR41, on immune, vascular, and adipose tissues, further contributing to an improved metabolic profile and reduced hypertension [53,56]. At this mechanistic intersection, the integrated pathways through which SGLT2i and probiotic interventions remodel gut microbiota, alter metabolite production, and activate host signaling cascades are summarized in Table 1.

In conclusion, the microbiota–SGLT2i–HF axis is characterized by a positive feedback loop: SGLT2is alter the gut environment, leading to favorable microbial shifts [64]; these shifts generate beneficial metabolites (increased SCFAs, reduced TMAO) and signaling molecules (enhanced secondary Bas) [37,40], which collectively enhance myocardial energy efficiency via βOHB and resolve systemic inflammation and endothelial dysfunction, thereby underpinning the clinical cardiorenal protection observed in diabetic HF [60,68]. The cumulative burden of dysbiosis in diabetic HF extends beyond systemic inflammation to direct myocardial toxicity. Gut-derived metabolites, particularly TMAO, have been shown to directly impair intracellular calcium signaling in cardiomyocytes, leading to reduced contractility and promoting structural remodeling of the heart [69]. These metabolic signals represent critical mechanistic nodes where microbiota-directed interventions could potentially synergize with SGLT2i therapy to mitigate the progression of diabetic cardiomyopathy.

## 6. Translational Implications and Future Directions: Towards Precision Cardiometabolic Intervention

The comprehensive mechanistic understanding of the microbiota–SGLT2i–HF axis elevates the gut microbiome from a mere pathological bystander to an integral and actionable therapeutic target in diabetic HF. This synthesis mandates a fundamental shift in clinical research priorities and informs novel strategies for precision medicine.

### 6.1. Microbiome Profiling for Enhanced Risk Stratification

The clear association between specific microbial signatures, such as SCFA producers and high TMA-producing taxa, and adverse cardiac outcomes suggests that microbiome profiling could serve as a non-invasive tool for enhanced risk stratification in patients with T2DM [70]. Recent prospective evidence highlights the clinical utility of measuring gut-derived uremic toxins; specifically, elevated serum indoxyl sulfate has been identified as a significant biomarker for aortic stiffness and vascular dysfunction in patients with T2DM [71]. The integration of metagenomics alongside clinical markers like NT-proBNP could identify high-risk patients [63]; however, the biogeographical distribution of these microbial signatures must be carefully considered when translating fecal profiles into clinical risk models [62]. This is particularly relevant given that the downstream vascular benefits, such as improved endothelial function and reduced arterial stiffness, are dependent on the systemic metabolic environment shaped by these microbes [68]. Furthermore, personalized assessment of plasma levels of gut-derived metabolites, specifically TMAO, may offer a dynamic window into the efficacy of SGLT2i intervention [40,68].

### 6.2. Multi-Omics Approaches and Causal Validation in HF Research

While correlational evidence is compelling, future research must prioritize multi-omics-driven investigations to establish causal relationships [63]. Large-scale studies are necessary to precisely map the signaling from the altered gut environment to the cardiomyocyte [66]. Advanced computational modeling is required for unraveling the complex feedback loops involving bile acid signaling pathways such as FXR and TGR5 [38,39], as well as the ketogenic effects driven by microbial activity [67]. Ultimately, validation requires germ-free animal models and targeted fecal microbiota transplantation (FMT) to establish causality [44]. Human cohort studies confirm that SGLT2i therapy reshapes the gut microbiome, enriching SCFA-producing bacteria such as *Faecalibacterium prausnitzii* [65].

### 6.3. Microbiota-Inclusive Therapeutic Strategies

Mechanistic data supports the development of microbiota-inclusive strategies that may synergize with SGLT2is. One promising area involves the targeted co-administration of prebiotics or synbiotics to amplify the enrichment of beneficial SCFA-producing bacteria [56,59]. Conversely, the targeted inhibition of TMA production represents a compelling strategy to eliminate a major source of cardiovascular toxicity [58]. Achieving maximal cardioprotection will require accounting for the complex biogeographical distribution of these microbial communities within the intestinal tract [62].

### 6.4. Challenges, Heterogeneity, Priorities, and Practical Barriers for Translating Microbiome Research

A major barrier to clinical translation of gut–heart axis findings is the high degree of heterogeneity across studies. Differences in dietary patterns, geographic and ancestral background, sample collection and fecal processing, sequencing platforms (16S rRNA versus shotgun metagenomics [66]), and bioinformatic pipelines produce inconsistent microbial signatures and limit cross-study comparability. Given these limitations, we have intentionally framed microbiome profiling and microbiota-inclusive therapies as research priorities rather than immediate clinical recommendations. Near-term priorities should include: (1) larger, multi-center longitudinal cohorts with repeated sampling and harmonized clinical phenotyping to define temporal relationships and prognostic value; (2) causal validation using randomized interventions, humanized animal models, and Mendelian or interventional approaches to move beyond association; (3) integration of multi-omics (metagenomics, metatranscriptomics, metabolomics, host transcriptomics) to map mechanistic pathways and identify reproducible biomarkers; and (4) development and adoption of standardized protocols for sample collection, storage, sequencing, and bioinformatic analysis to reduce methodological heterogeneity. Practical barriers to implementation must also be addressed, including assay validation and quality control, regulatory and reimbursement pathways for microbiome diagnostics and therapeutics, cost and logistical constraints for routine sampling, and the need for geographically and ancestrally diverse cohorts to ensure generalizability. By prioritizing these steps, the field can responsibly evaluate whether microbiome profiling or adjunctive microbiota-directed therapies can be translated into safe, effective, and equitable clinical tools.

A significant challenge in translating gut–heart axis research into clinical practice is the high degree of heterogeneity across studies. Variations in dietary patterns, ethnic backgrounds, and fecal processing techniques often lead to inconsistent microbial signatures. Furthermore, the lack of standardized bioinformatic pipelines and the reliance on 16S rRNA sequencing versus metagenomic shotgun sequencing contribute to disparate results across different cohorts [66]. These factors necessitate a cautious interpretation of current findings until more standardized, multi-center longitudinal studies are conducted.

## 7. Conclusions

The microbiota–SGLT2i–HF axis may represent a promising frontier for precision medicine. Current evidence is preliminary, and clinical translation requires multi-omics validation and longitudinal human studies. This synthesis aims to stimulate further investigation rather than propose definitive therapeutic recommendations.

The profound cardiorenal protection offered by SGLT2is is inextricably linked to a systemic signaling cascade initiated within the gut. SGLT2i therapy has been associated in preclinical and small clinical studies with shifts in gut microbial composition that may favor SCFA-producing taxa, driven by increased luminal glucose concentration. This environment selects for beneficial taxa, notably enriching SCFA producers such as *Akkermansia muciniphila* [42], while suppressing pathobionts responsible for generating harmful metabolites such as TMA. The resulting shift in the microbial metabolome directly targets Hf pathology through several integrated pathways that function as a cohesive metabolic network. Central to this process is the establishment of an SCFA-rich environment, which synergizes with systemic metabolic shifts to elevate βOHB levels, thereby providing the failing myocardium with a more efficient fuel source [67]. This energetic enhancement occurs in parallel with a significant reduction in systemic toxicity, as the suppression of TMA-producing flora leads to a measurable decrease in circulating TMAO, subsequently mitigating endothelial dysfunction and vascular stress [69]. Furthermore, the modulation of the bile acid pool serves as a critical regulatory node by activating the FXR and TGR5. This molecular activation strengthens the intestinal barrier and dampens the chronic, low-grade inflammation [38] that fundamentally drives adverse cardiac remodeling. By integrating these energetic, antitoxic, and anti-inflammatory mechanisms, the gut–heart axis establishes a multifaceted defense against the progression of diabetic HF.

Ultimately, the microbiota–SGLT2i–HF axis represents a promising frontier for precision medicine, allowing for enhanced risk stratification and personalized, microbiota-inclusive therapeutic strategies.

**Figure 1:**
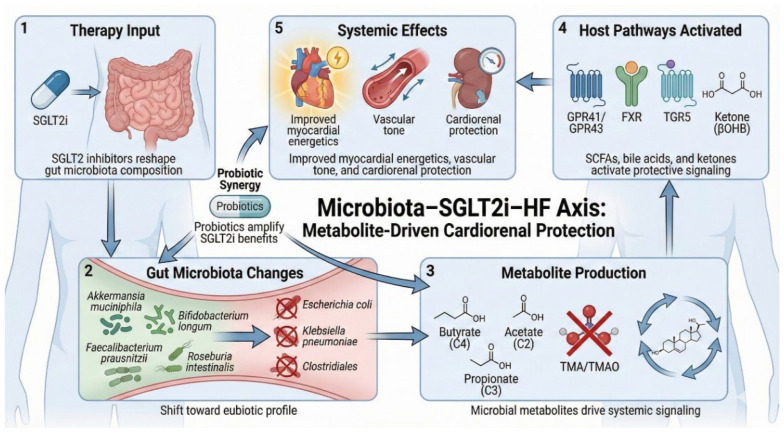
**The microbiota–SGLT2i–HF axis.** A five-panel schematic illustrating how SGLT2i therapy may modulate the gut ecosystem and produce downstream cardiometabolic benefits. (**Panel 1**) Oral SGLT2 inhibitor entering the gastrointestinal tract with reduced luminal glucose availability. (**Panel 2**) A shift toward eubiosis, highlighting the proliferation of beneficial bacteria (*Akkermansia muciniphila*, *Bifidobacterium longum*, *Faecalibacterium prausnitzii*, *Roseburia intestinalis*) and the suppression of pathobionts (*Escherichia coli*, *Klebsiella pneumoniae*, Clostridiales). (**Panel 3**) Metabolite profile changes, including increased SCFAs, e.g., butyrate, acetate, and propionate, altered bile acid conversion, and reduced trimethylamine N-oxide (TMAO). (**Panel 4**) Host signaling with SCFA ligands engaging GPR41/GPR43 bile acids activating FXR and TGR5 and increasing ketones, e.g., beta hydroxybutyrate. (**Panel 5**) Systemic phenotypic outcomes, including improved myocardial energetics, improved vascular tone, and reduced renal stress. (A probiotic capsule is illustrated as an adjuvant therapy) Adjuvant microbiome-directed interventions such as targeted probiotics/prebiotics feeding back to reinforce eubiosis.

In summary, the microbiota–SGLT2i–HF axis functions as a positive feedback loop wherein drug administration favorably remodels the gut ecosystem, resulting in a cascade of beneficial metabolites and signaling molecules that directly resolve the metabolic and inflammatory hallmarks of diabetic HF. This emerging understanding paves the way for future precision cardiometabolic interventions, including microbiome profiling for risk stratification and the co-administration of targeted prebiotics or synbiotics, to maximize the cardioprotective efficacy of SGLT2i therapy. A five-panel schematic diagram in Figure 1 illustrates the circular mechanism of the microbiota–SGLT2i–HF axis. Panel 1 depicts an SGLT2 inhibitor capsule entering the gastrointestinal tract, indicating the initiation of gut modulation. Panel 2 shows a shift toward eubiosis, highlighting the proliferation of beneficial bacteria (*Akkermansia muciniphila*, *Bifidobacterium longum*, *Faecalibacterium prausnitzii*, and *Roseburia intestinalis*) and the suppression of pathobionts (*Escherichia coli*, *Klebsiella pneumoniae*, and Clostridiales). Panel 3 illustrates the resulting metabolite profile: increased production of SCFAs like butyrate, acetate, and propionate; optimized bile acid conversion; and the explicit reduction of toxic TMAO. Panel 4 demonstrates the activation of host signaling pathways, showing SCFA ligands binding to GPR41/GPR43 receptors, bile acids activating FXR/TGR5, and the presence of β-hydroxybutyrate (βOHB). Panel 5 displays the systemic phenotypic outcomes: a heart with improved energetics, a dilated vessel indicating improved vascular tone, and a kidney representing reduced renal stress. A probiotic capsule is illustrated as an adjuvant therapy.

A notable limitation of this review is that the literature search was restricted exclusively to the PubMed and ScienceDirect databases. The exclusion of other prominent repositories such as Embase or the Cochrane Library may have resulted in the omission of relevant clinical and translational studies, thereby limiting the comprehensive capture of all global evidence pertaining to this therapeutic axis.

## Figures and Tables

**Table 1:** Integrated microbiota–SGLT2i—heart failure axis. The convergent pathways through which SGLT2i and probiotic interventions remodel the gut microbiota, alter metabolite production, and activate host signaling cascades. Enrichment of SCFA-producing taxa and suppression of pathobionts reduce TMAO and LPS, while modulation of bile acid pools enhances FXR and TGR5 activity. These changes collectively improve myocardial energetics via βOHB, strengthen endothelial function, and attenuate systemic inflammation, thereby supporting the cardiorenal protection observed in diabetic heart failure.

Mechanistic Axis	Microbiota or Probiotic Modulation	Key Metabolites or Pathways	Impact on Heart Failure	References
**The TMAO–Atherosclerosis Axis**	Enrichment of choline-metabolizing bacteria (e.g., *Clostridiales*, *Proteobacteria*).	↑ TMAO; hepatic FMO3 activation.	Promotes endothelial dysfunction, vascular stiffness, and myocardial fibrosis; predicts adverse HF events.	[46,47,54,55,59]
**The SGLT2i–Eubiosis Axis**	SGLT2i-induced glycosuria selects for SCFA producers (*Akkermansia*, *Faecalibacterium*, *Bifidobacterium*); suppresses pathobionts (*Klebsiella*, *E. coli*).	↑ SCFAs (butyrate, acetate, propionate); ↓ uremic toxins (Indoxyl sulfate).	Improves endothelial function via GPR41/43; reduces systemic inflammation and oxidative stress.	[37,42,48,49,60,64]
**The Ketogenic–Bioenergetic Axis**	Enrichment of butyrate-producing taxa (*Roseburia intestinalis*) synergizes with SGLT2i pharmacology.	↑ βOHB; hepatic ketogenesis fueled by gut-derived butyrate.	Provides “thrifty fuel” for the energy-starved myocardium; enhances mitochondrial biogenesis and repair.	[62,63,66]
**The Bile Acid–Nuclear Receptor Axis**	Modulation of bile acid-transforming bacteria; SGLT2i-mediated alteration of the bile acid pool.	↑ Secondary bile acids; activation of FXR and TGR5 receptors.	Promotes intestinal barrier integrity; regulates anti-inflammatory effects on immune and vascular cells.	[38,39,40]
**The Endotoxemia–Inflammation Axis**	Dysbiosis-induced “Leaky Gut”; loss of mucin-degrading bacteria (*A. muciniphila*).	↑ LPS; activation of TLR4 signaling.	Essential chronic low-grade inflammation; promotes insulin resistance and diabetic cardiomyopathy remodeling.	[41,42,45]
**Therapeutic Probiotic Synergy**	*Lactobacillus rhamnosus*, *Bifidobacterium longum*, or specific butyrate producers.	Restoration of SCFA levels; inhibition of TMA lyases.	Supports amplification of SGLT2i benefits; improves post-infarction remodeling.	[60,64,66]

βOHB: β-hydroxybutyrate; FMO3: Flavin-containing monooxygenase 3; FXR: Farnesoid X Receptor; GPR41/43: G protein-coupled receptor 41/43; HF: Heart Failure; LPS: Lipopolysaccharide; OXPHOS: Oxidative Phosphorylation; PGC-1α: Peroxisome proliferator-activated receptor-gamma coactivator 1-alpha; SCFA: Short-Chain Fatty Acid; SGLT2i: Sodium-Glucose Cotransporter 2 inhibitor; TGR5: Takeda G protein-coupled receptor 5; TLR4: Toll-like Receptor 4; TMA: Trimethylamine; TMAO: Trimethylamine N-oxide; ↑: increase; ↓: decrease.

## Data Availability

No new data were created or analyzed in this study. Data sharing is not applicable to this article.

## Supplementary Materials

The following supporting information can be downloaded at: https://www.mdpi.com/article/10.3390/ijms27094101/s1.

## Abbreviations

βOHB: beta hydroxybutyrate; FXR: farnesoid X receptor; GPR41/43: G protein-coupled receptor 41/43. SCFA: short-chain fatty acid; SGLT2i: sodium glucose cotransporter 2 inhibitor; TGR5: G protein bile acid receptor; TMAO: trimethylamine N-oxide.

## References

[1] Gonzalez Manzanares R., Anguita Gámez M., Muñiz J., Gimeno-Orna J.A., Pérez A., Rodríguez-Padial L., Anguita M. (2024). DIABETIC-IC study Investigators. Prevalence and incidence of heart failure in type 2 diabetes patients: Results from a nationalwide prospective cohort—The DIABET-IC study. Cardiovasc. Diabetol..

[2] Thal S.C., Shityakov S., Salvador E., Förster C.Y. (2025). Heart rate variability, microvascular dysfunction, and inflammation: Exploring the potential of taVNS in managing heart failure in type 2 diabetes mellitus. Biomolecules.

[3] Yang D.R., Wang M.Y., Zhang C.L., Wang Y. (2024). Endothelial dysfunction in vascular complications of diabetes: A comprehensive review of mechanisms and implications. Front. Endocrinol..

[4] Ceriello A., Catrinoiu D., Chandramouli C., Cosentino F., Dombrowsky A.C., Itzhak B., Lalic N.M., Prattichizzo F., Schnell O., Seferović P.M. (2021). Heart failure in type 2 diabetes: Current perspectives on screening, diagnosis and management. Cardiovasc. Diabetol..

[5] Cheng C., Liu Y., Sun L., Fan J., Sun X., Zheng J.S., Zheng L., Zhu Y., Zhou D. (2025). Integrative metabolomics and genomics reveal molecular signatures for type 2 diabetes and its cardiovascular complications. Cardiovasc. Diabetol..

[6] Nesti L., Pugliese N.R., Sciuto P., Natali A. (2020). Type 2 diabetes and reduced exercise tolerance: A review of the literature through an integrated physiology approach. Cardiovasc. Diabetol..

[7] Vaduganathan M., Docherty K.F., Claggett B.L., Jhund P.S., de Boer R.A., Hernandez A.F., Inzucchi S.E., Kosiborod M.N., Lam C.S.P., Martinez F. (2022). SGLT-2 inhibitors in patients with heart failure: A comprehensive meta-analysis of five randomized controlled trials. Lancet.

[8] Li F., Baheti R., Jin M., Xiong W., Duan J., Fang P., Wan J. (2024). Impact of SGLT2 inhibitors on cardiovascular outcomes and metabolic events in Chinese han patients with chronic heart failure. Diabetol. Metab. Syndr..

[9] Zheng X.D., Qu Q., Jiang X.Y., Wang Z.Y., Tang C., Sun J.Y. (2021). Effects of dapagliflozin on cardiovascular events, death, and safety outcomes in patients with heart failure: A meta-analysis. Am. J. Cardiovasc. Drugs.

[10] Yu X., Zhao L., Liu H., Zhou X., Zhao G., Zhang Z., Qian X., Sun B., Fang S., Yang Q. (2025). SGLT2 Inhibitors and improved survival in patients with diabetes and acute myocardial infarction: Evidence from an electronic health record-based cohort study. Am. J. Cardiovasc. Drugs.

[11] Hatamnejad M.R., Medzikovic L., Dehghanitafti A., Rahman B., Vadgama A., Eghbali M. (2025). Role of gut microbial metabolites in ischemic and non-ischemic heart failure. Int. J. Mol. Sci..

[12] Li C., Stražar M., Mohamed A.M.T., Pacheco J.A., Walker R.L., Lebar T., Zhao S., Lockart J., Dame A., Thurimella K. (2024). Gut Microbiome and metabolome Profiling in Framingham Heart Study reveals cholesterol-metabolizing bacteria. Cell.

[13] Bui T.V.A., Hwangbo H., Lai Y., Hong S.B., Choi Y.-J., Park H.-J., Ban K. (2023). The Gut-Heart Axis: Updated Review for The Roles of Microbiome in Cardiovascular Health. Korean Circ. J..

[14] Chen H.C., Liu Y.W., Chang K.C., Wu Y.W., Chen Y.M., Chao Y.K., You M.Y., Lundy D.J., Lin C.J., Hsieh M.L. (2023). Gut butyrate-producers confer post-infarction cardiac protection. Nat. Commun..

[15] Gąsecka A., Gniot M., Rajewska B., Dykacz W., Kisielewska W., Błażejowska E., Zimodro J.M., Grabowski M., Rymuza B., Huczek Z. (2025). Transcatheter aortic valve implantation reduces plasma concentrations of TMAO and indoxyl sulfate: A prospective, multicenter cohort study. Cardiol. J..

[16] Song G., Xie Y., Yi L., Cheng W., Jia H., Shi W., Liu Q., Fang L., Xue S., Liu D. (2025). Bile acids affect intestinal barrier function through FXR and TGR5. Front. Med..

[17] Xi Y., Chen D., Dong Z., Zhang J., Lam H., He J., Du K., Chen C., Guo J., Xiao J. (2022). Multi-omics insights into potential mechanism of SGLT2 inhibitors cardiovascular benefit in diabetic cardiomyopathy. Front. Cardiovasc. Med..

[18] Zou B., Huo Q., Zhou X., Lv Y., Li G., Fu G., Shen H., Shu S. (2025). Characteristics and longitudinal stability of gut microbiota in healthy individuals across different age groups. Curr. Res. Microb. Sci..

[19] Han Y., Wang Z., Xie J., Yang G., Su M., Wang S., Yang M., Yu H., Li M., Wang L. (2026). Host-gut microbiota interactions in health and disease: Mechanisms and intervention strategies. Front. Microbiol..

[20] Almeida C., Gonçalves-Nobre J.G., Costa D.A., Barata P. (2023). The potential links between human gut microbiota and cardiovascular health and disease-is there a gut-cardiovascular axis?. Front. Gastroenterol..

[21] Zhu J., Lyu J., Zhao R., Wang S. (2023). Gut macrobiotic and its metabolic pathways modulate cardiovascular disease. Front. Microbiol..

[22] Zhang Y., Wu H., Jin M., Feng G., Wang S. (2025). The gut-heart axis: Unveiling the roles of gut microbiota in cardiovascular diseases. Front. Cardiovasc. Med..

[23] Yang C., Li X., Hu M., Li T., Jiang L., Zhang Y. (2024). Gut Microbiota as Predictive Biomarker for Chronic Heart Failure in Patients with Different Nutritional Risk. J. Cardiovasc. Transl. Res..

[24] Albulushi A., Taha T. (2025). Gut microbiome dysbiosis in heart failure: Updated evidence, mechanisms, and therapeutic directions. Am. Heart J. Plus..

[25] Shi H., Wu M., Wu X., Liu Z., Jiang S., Li G., Yang Y., Fu Y., Wang Q., Zhang G. (2025). Multi-omics integration reveals functional signatures of gut microbiome in atherosclerosis. Gut Microbes.

[26] Johnson S.A., Weir T.L. (2024). Gut microbiome-derived secondary bile acids: Therapeutic targets for reducing cardiovascular disease in type 2 diabetes?. Am. J. Clin. Nutr..

[27] Rezabakhsh A., Habtemariam S., Parvizi R., Meddahi-Pellé A., Ruiz V.R., Pavon-Djavid G., Barzgari A. (2025). The gut-heart axis: A correlation between Paneth cells’ dysfunction, microbiome dysbiosis, and cardiovascular diseases. Cell. Commun. Signal..

[28] Modrego J., Ortega-Hernández A., Goirigolzarri J., Restrepo-Córdoba M.A., Bäuerl C., Cortés-Macías E., Sánchez-González S., Esteban-Fernández A., Pérez-Villacastín J., Collado M.C. (2023). Gut Microbiota and Derived Short-Chain Fatty Acids Are Linked to Evolution of Heart Failure Patients. Int. J. Mol. Sci..

[29] Zhou S., Zhou X., Zhang P., Zhang W., Huang J., Jia X., He X., Sun X., Su H. (2025). The gut microbiota-inflammation-HfpEF axis: Deciphering the role of gut microbiota dysregulation in the pathogenesis and management of HfpEF. Front. Cell. Infect. Microbiol..

[30] Chulenbayeva L., Issilbayeva A., Sailybayeva A., Bekbossynova M., Kozhakhmetov S., Kushugulova A. (2025). Short-Chain Fatty Acids and Their Metabolic Interactions in Heart Failure. Biomedicines.

[31] Lu Q., Chen J., Jiang L., Geng T., Tian S., Liao Y., Yang K., Zheng Y., He M., Tang H. (2024). Gut microbiota-derived secondary bile acids, bile acid receptor polymorphisms, and risk of cardiovascular disease in individuals with newly diagnosed type 2 diabetes: A cohort study. Am. J. Clin. Nutr..

[32] Wu H., Esteve E., Tremaroli V., Khan M.T., Caesar R., Mannerås-Holm L., Ståhlman M., Olsson L.M., Serino M., Planas-Fèlix M. (2017). Metformin alters the gut microbiome of individuals with treatment-naive type 2 diabetes, contributing to the therapeutic effects of the drug. Nat. Med..

[33] Wu M., Zhou X., Chen S., Wang Y., Lu B., Zhang A., Zhu Y., Huang M., Wang J., Liu J. (2025). The alternations of gut microbiota in diabetic kidney disease: Insights from a triple comparative cohort. Front. Cell. Infect. Microbiol..

[34] Mishra S., Jain S., Agadzi B., Yadav H. (2025). A cascade of microbiota–leaky gut–inflammation: Is it a key player in metabolic disorders?. Curr. Obes. Rep..

[35] Chen B., Gautron L. (2025). Gut-derived lipopolysaccharides and metabolic endotoxemia: A critical review. Am. J. Physiol. Endocrinol. Metab..

[36] Liu J., Li F., Yang L., Luo S., Deng Y. (2025). Gut microbiota and its metabolites regulate insulin resistance: Traditional Chinese medicine insights for T2DM. Front. Microbiol..

[37] Zeng L., Ma J., Wei T., Wang H., Yang G., Han C., Zhu T., Tian H., Zhang M. (2024). The effect of canagliflozin on gut microbiota and metabolites in type 2 diabetic mice. Genes Genom..

[38] R. Muralitharan R., Zheng T., Dinakis E., Xie L., Barbaro-Wahl A., Jama H.A., Nakai M., Paterson M., Leung K.C., McArdle Z. (2025). Gut microbiota metabolites sensed by host GPR41/43 protect against hypertension. Circ. Res..

[39] Qi Z., Zhang W., Zhang P., Qu Y., Zhong H., Zhou L., Zhou W., Yang W., Xu H., Zhao X. (2025). The gut microbiota-bile acid-TGR5 axis orchestrates platelet activation and atherothrombosis. Nat. Cardiovasc. Res..

[40] Deng X., Zhang C., Wang P., Wei W., Shi X., Wang P., Yang J., Wang L., Tang S., Fang Y. (2022). Cardiovascular benefits of empagliflozin are associated with gut microbiota and plasma metabolites in Type 2 diabetes. J. Clin. Endocrinol. Metab..

[41] Liu Y., Li X., Chen Y., Yao Q., Zhou J., Wang X., Meng Q., Ji J., Yu Z., Chen X. (2025). Fecal microbiota transplantation: Application scenarios, efficacy prediction, and factors impacting donor-recipient interplay. Front. Microbiol..

[42] Panzetta M.E., Valdivia R.H. (2024). Akkermansia in the gastrointestinal tract as a modifier of human health. Gut Microbes.

[43] Wang L., Wang Y., Xu H., Li W. (2024). Effect of dapagliflozin on ferroptosis through the gut microbiota metabolite TMAO during myocardial ischemia-reperfusion injury in diabetes mellitus rats. Sci. Rep..

[44] Lee D.M., Battson M.L., Jarrell D.K., Hou S., Ecton K.E., Weir T.L., Gentile C.L. (2018). SGLT2 inhibition via dapagliflozin improves generalized vascular dysfunction and alters the gut microbiota in type 2 diabetic mice. Cardiovasc. Diabetol..

[45] Olędzki S., Kukowka A., Siennicka A., Jakubiak N., Maciejewska-Markiewicz D., Kiedrowicz R., Kaźmierczak J. (2025). Serum zonulin and lipopolysaccharide (LPS) levels in early myocardial infarction: Association with left ventricular ejection fraction. J. Clin. Med..

[46] Yu X., Wang Y., Yang R., Wang Z., Wang X., Wang S., Zhang W., Dong J., Chen W., Ji F. (2024). Trimethylamine N-oxide predicts cardiovascular events in coronary artery disease patients with diabetes mellitus: A prospective cohort study. Front. Endocrinol..

[47] Tsai T.Y., Aldujeli A., Haq A., Murphy P., Unikas R., Sharif F., Garg S., Brilakis E.S., Onuma Y., Serruys P.W. (2025). Trimethylamine N-Oxide as a Biomarker for Left Ventricular Diastolic Dysfunction and Functional Remodeling After STEMI. Int. J. Mol. Sci..

[48] GóesSantos B.R., Castro P.C., Girardi A.C.C., Antunes-Correa L.M., Davel A.P. (2025). Vascular effects of SGLT2 inhibitors: Evidence and mechanisms. Am. J. Physiol. Cell Physiol..

[49] Mindrescu N.M., Guja C., Jinga V., Ispas S., Curici A., Danciulescu Miulescu R.E., Twakor A.N., Stoian A.M.P. (2025). SGLT2 inhibitors and metabolic outcomes: A primary data study exploring the microbiota–diabetes connection. Metabolites.

[50] Karlsson F.H., Tremaroli V., Nookaew I., Bergström G., Behre C.J., Fagerberg B., Nielsen J., Bäckhed F. (2013). Gut metagenome in European women with normal, impaired and diabetic glucose control. Nature.

[51] Li Z., Wu Z., Yan J., Liu H., Liu Q., Deng Y., Ou C., Chen M. (2019). Gut microbe-derived metabolite trimethylamine N-oxide induces cardiac hypertrophy and fibrosis. Lab. Investig..

[52] Qin J., Li Y., Cai Z., Li S., Zhu J., Zhang F., Liang S., Zhang W., Guan Y., Shen D. (2012). A metagenome-wide association study of gut microbiota in type 2 diabetes. Nature.

[53] Chen L., He F.J., Dong Y., Huang Y., Wang C., Harshfield G.A., Zhu H. (2020). Modest Sodium Reduction Increases Circulating Short-Chain Fatty Acids in Untreated Hypertensives: A Randomized, Double-Blind, Placebo-Controlled Trial. Hypertension.

[54] Chen M.L., Zhu X.H., Ran L., Lang H.D., Yi L., Mi M.T. (2017). Trimethylamine-N-Oxide induces vascular inflammation by activating the NLRP3 inflammasome through the SIRT3-SOD2-mtROS signaling pathway. J. Am. Heart Assoc..

[55] Li X., Geng J., Zhao J., Ni Q., Zhao C., Zheng Y., Chen X., Wang L. (2019). Trimethylamine NOxide exacerbates cardiac fibrosis via activating the NLRP3 inflammasome. Front. Physiol..

[56] Bartolomaeus H., Balogh A., Yakoub M., Homann S., Markó L., Höges S., Tsvetkov D., Krannich A., Wundersitz S., Avery E.G. (2019). Short-chain fatty acid propionate protects from hypertensive cardiovascular damage. Circulation.

[57] Tang W.H., Wang Z., Levison B.S., Koeth R.A., Britt E.B., Fu X., Wu Y., Hazen S.L. (2013). Intestinal microbial metabolism of phosphatidylcholine and cardiovascular risk. N. Engl. J. Med..

[58] McMurray J.J.V., Solomon S.D., Inzucchi S.E., Køber L., Kosiborod M.N., Martinez F.A., Ponikowski P., Sabatine M.S., Anand I.S., Bělohlávek J. (2019). Dapagliflozin in patients with heart failure and reduced ejection fraction. N. Engl. J. Med..

[59] Jaworska K., Kuś M., Ufnal M. (2025). TMAO and diabetes: From the gut feeling to the heart of the problem. Nutr. Diabetes.

[60] Tao Y., Zhang N., Wang Z., Pan Y., Zhong S., Liu H. (2025). SGLT2 inhibitors confer cardiovascular protection via the gut-kidney-heart axis: Mechanisms and translational perspectives. J. Cardiovasc. Dev. Dis..

[61] Sperry B.W., Almohtasib Y., Ghaly R., Abdel Jawad M., Sauer A.J., Bateman T.M. (2025). SGLT2 inhibitor treatment is associated with reduced cardiac glucose metabolism: A matched FDG-PET cohort study. JACC Adv..

[62] Yang J.C., Lagishetty V., Aja E., Arias-Jayo N., Chang C., Hauer M., Katzka W., Zhou Y., Sedighian F., Koletic C. (2025). Biogeographical distribution of gut microbiome composition and function is partially recapitulated by fecal transplantation into germ-free mice. ISME J..

[63] Yuan F., Zhang T., Jia S., Zhao J., Wan B., Liu G. (2024). Fine mapping-based multi-omics analysis interprets the gut-lung axis function of SGLT2 inhibitors. Front. Cell. Infect. Microbiol..

[64] Kanbay M., Al-Shiab R., Shah E., Ozbek L., Guldan M., Ortiz A., Fouque D. (2025). Gut microbiota modulation in GLP-1RA and SGLT2i therapy: Clinical implications and mechanistic insights in type 2 diabetes. Clin. Kidney J..

[65] Kusunoki M., Hisano F., Matsuda S.I., Kusunoki A., Wakazono N., Tsutsumi K., Miyata T. (2023). Effects of SGLT2 inhibitors on the intestinal bacterial flora in Japanese patients with type 2 diabetes mellitus. Drug Res..

[66] Nagatomo Y., Tang W.H. (2015). Intersections between microbiome and heart failure: Revisiting the gut hypothesis. J. Card. Fail..

[67] Su S., Ji X., Li T., Teng Y., Wang B., Han X., Zhao M. (2023). The changes of cardiac energy metabolism with sodium glucose transporter 2 inhibitor therapy. Front. Cardiovasc. Med..

[68] Polidori D., Sha S., Mudaliar S., Ciaraldi T.P., Ghosh A., Vaccaro N., Farrell K., Rothenberg P., Henry R.R. (2013). Canagliflozin lowers postprandial glucose and insulin by delaying intestinal glucose absorption in addition to increasing urinary glucose excretion: Results of a randomized, placebo-controlled study. Diabetes Care.

[69] Savi M., Bocchi L., Bresciani L., Falco A., Quaini F., Mena P., Brighenti F., Crozier A., Stilli D., Del Rio D. (2018). Trimethylamine-N-oxide (TMAO)-induced impairment of cardiomyocyte function and the protective role of urolithin B-glucuronide. Molecules.

[70] Wang Z., Klipfell E., Bennett B.J., Koeth R., Levison B.S., Dugar B., Feldstein A.E., Britt E.B., Fu X., Chung Y.M. (2011). Gut flora metabolism of phosphatidylcholine promotes cardiovascular disease. Nature.

[71] Su I.M., Teng Y.Y., Li J.C., Liu C.H., Wu D.A., Hsu B.G. (2025). Serum indoxyl sulfate as a potential biomarker of aortic stiffness in persons with type 2 diabetes mellitus. Medicina.

