# Supplementary material for: Targeting the Gut–Heart Axis in Diabetic Heart Failure: Microbiota and SGLT2is as Converging Therapeutic Frontiers
Source: Int J Mol Sci. 2026 May 3;27(9):4101. doi: 10.3390/ijms27094101 (PMC13164356; doi:10.3390/ijms27094101)
Supplement: Supplementary file 1 [file ijms-27-04101-s001.zip › ijms-4245228-supplementary.pdf]

Table S1. Characteristics and key findings of principal studies included in the review.

Reference	Study type	Population or model	Key microbiota or metabolite finding	Major limitation
[29]	Cohort or mechanistic review study	Studies linking gut inflammation to HFpEF	Provided rationale for phenotype specific microbiota effects relevant to HFpEF	Lacks direct SGLT2i microbiome comparisons by heart failure phenotype
[37]	Preclinical study	Murine model of SGLT2 inhibition	Reported SGLT2i associated shifts in microbial composition and bile acid profiles	Animal model with limited translatability to humans
[40]	Small human cohort	Patients treated with empagliflozin or metformin (n=36)	Observed changes in plasma metabolites and modest microbiota shifts	Small sample size and short follow up
[58]	Large randomized clinical trial	HFrEF population treated with dapagliflozin (n=386)	Demonstrated clinical benefit of SGLT2i in HFrEF supporting phenotype distinction	Did not include microbiome or metabolite endpoints
[61]	Human mechanistic study	Clinical cohort assessing cardiac metabolism (n=187)	Documented metabolic effects of SGLT2i relevant to myocardial energetics, indirect for microbiota	Focused on cardiac metabolism rather than microbiome endpoints
[62]	Multicohort	Geographically	Highlighted	Heterogeneous

Reference	Study type	Population or model	Key microbiota or metabolite finding	Major limitation
	analysis or review	diverse cohorts	biogeographical variability in microbiome signatures relevant to cardiometabolic disease	methods across cohorts limit comparability
[64]	Clinical review and small studies synthesis	Mixed clinical and preclinical evidence	Discussed microbiota modulation by SGLT2i and related agents	Narrative synthesis without formal meta analysis
[65]	Small clinical study	Single center human cohort (n=36)	Reported enrichment of putative SCFA producing taxa after SGLT2i exposure	Single center small cohort requiring replication
[66]	Review on microbiome and heart failure	Literature synthesis	Emphasized methodological heterogeneity and need for standardized approaches	Broad scope and not primary data

n denotes the number of enrolled subjects in the study.
